# The future of medical physics in the US health‐care system

**DOI:** 10.1120/jacmp.v14i5.4622

**Published:** 2013-09-06

This issue, I would like to digress from the meaning of the *JACMP* and discuss with you some personal thoughts on the US health‐care system and the future of medical physics under the *Affordable Care Act*. My credentials for discussing this topic include a newly minted PhD in Health Management from the School of Public Health at the University of Louisville. I spent many rewarding hours in classes, seminars, and lectures learning about the changes in US health care and public health over the past 11 years. This is a very difficult topic to explore in the limited space assigned to an editorial, but I would like to point out some themes that we need to bear in mind as we look forward.

1. Efficiency — It is expected that the *Affordable Care Act* will add approximately 30 million people to the roles of the insured. Health‐care workers likely will be expected to absorb the associated additional work with only modest increases in employment. Please keep in mind that hundreds of billions of dollars are being diverted from the Medicare Program over the next decade to fund the services provided to these additional patients and consumers. Now add to this dynamic the large number of retiring baby‐boom health‐care workers and the loss of a large base of knowledge and experience. It is projected that up to 30% of the health‐care workforce could retire within the next five years. While younger workers are usually less expensive to providers, they also may be less efficient in the delivery of quality services. Those health delivery systems that can deliver health services at community standard of care quality will be at a competitive advantage. For example, if a cancer center can treat patients presenting with a certain disease with fewer fractions than another while reporting similar and competitive outcomes, the gatekeepers of the systems likely will seek out and reward a more efficient provider.
2. Training — Barriers are increasing for those seeking to enter the heath‐care workforce. Medical physics MS programs are charging up to $20,000 per year for tuition. Student loans for this training are running at a 7.5% annual interest rate. Financing a medical school education is even more challenging. If a student borrows $200,000 to go to medical school, then moves on to complete a residency program, it may be years before a young physician can begin to make a dent in this liability. Yes, some physicians earn significant financial rewards, but such success is by no means guaranteed. Physicians of tomorrow may very well face student loan debt larger than their mortgages. Although medical physics training pathways are shorter and less expensive, financial barriers are both significant and growing.
3. Reimbursement — If you look at business trends, health‐care providers are merging into enormous entities composed of multiple hospitals and clinics. It may be that one primary purpose for this trend is to become large enough to negotiate directly with the primary payers. It is no secret the Center for Medicare and Medicaid Services (CMS) seems to be moving away from the Current Procedural Terminology (CPT)‐based fee‐for‐service reimbursement for outpatient health services. Each year, we see more services being bundled under the broader Ambulatory Patient Classification (APC) categories, with the apparent ultimate goal seeming to be to assign a dollar value for each International Classification of Disease (ICD‐9 or ICD‐10) patient diagnosis. There are dozens of ICD codes for breast cancer (just one example), and most other major cancers you can name. Once the relative values of each code are assigned, it will be possible to name a single multiplier to the entire table to determine the reimbursement of any patient presentation. The multiplier would be determined by the funds available and political realities. This approach could replace the current fee‐for‐service reimbursement system. The bottom line is that the value of work performed by medical physicists could easily get lost in the consolidation of the reimbursement and payment system, along with the trend of health‐care providers merging into larger and even larger entities. We will need to redouble our efforts to be visible and relevant.
4. Competition — For example, a large not‐for‐profit US hospital chain and a hospital chain from an Asian nation together are building a hospital in the Cayman Islands. Large investment banks fund this multibillion dollar project. Ultimately the plans include a two thousand‐bed hospital, medical school, research center, as well as a biotechnology park and assisted living complex. It intends to seek Joint Commission International Accreditation. This is only one of many such projects either being constructed or planned in the Caribbean and Central America. The funding for such projects comes from those that anticipate there will be many willing to travel for quality services offered at lower cost. Some aspects of the Cayman Islands project include the importation of health‐care professionals from other countries with no additional requirements to practice and no taxes on imported capital equipment, and the purchase of equipment and supplies at the rates that hospital groups in Asia pay. Considering the proximity of the Cayman Islands to Florida, Texas, and the other Gulf Coast states, it seems very likely to me those 2,000 beds might not be enough.

My conclusion from what I have seen in the literature and heard from my instructors is the US health‐care system will undergo enormous changes over the next few years. These will be times of uncertainty and struggle for all health professions. Although medical physicists are in some ways better equipped to weather the storm than others, we too might face significant pressures. Are we ready for the challenge?

Michael D. Mills

Editor‐in‐Chief

